# Treatment of type 2 diabetes and stress using neuro-emotional technique: case report

**DOI:** 10.3389/fendo.2024.1382757

**Published:** 2024-07-10

**Authors:** Peter Bablis, Ryan R. Day, Henry Pollard

**Affiliations:** ^1^ Department of Maternal and Child Health and Precision Medicine, University Research Institute, Athens, Greece; ^2^ Department of Integrative and Complementary Medicine, Universal Health, Sydney, NSW, Australia; ^3^ Faculty of Health Sciences, Durban University of Technology, Durban, South Africa

**Keywords:** type 2 diabetes (T2D), neuro-emotional technique (NET), psycho-immune-neuroendocrine (PINE) network, adverse childhood experiences (ACEs), allostatic load, stress

## Abstract

This case report presents a novel, non-pharmacological treatment of Type 2 Diabetes in a 46-year-old male, demonstrating improvements in blood chemistry and psychometric markers after 8 treatments using a Mind-Body Intervention (MBI) called Neuro-Emotional Technique (NET). The patient presented with a diagnosis of Type 2 Diabetes (T2D), pain, psychosocial indicators of stress and anxiety, and a score of 4 on the ACE-Q (Adverse Childhood Experiences Questionnaire) that is consistent with a predisposition to chronic disease and autoimmune disorders. Glucose levels for this patient were above normal levels (typically between 10-15mmol/L where optimal range is between 4-10mmol/L) for at least two months prior to the 4-week NET intervention period, despite the standard use of conventional antidiabetic medications (insulin injections). The patient exhibited numerous indictors of chronic stress that were hypothesised to be underlying his medical diagnosis and a series of 8 NET treatments over a period of 4 weeks was recommended. Psychometric tests and glucose measurements were recorded at baseline (prior to treatment), 4 weeks (at the conclusion of treatment) and at 8 weeks (4 weeks following the conclusion of treatment). Results show that glucose levels were reduced, and self-reported measures of depression, anxiety, stress, distress and pain all decreased from high and extreme levels to within normal ranges after 4 weeks, with ongoing improvement at 8 weeks. McEwen described the concept of allostatic load and the disruptive effects that cumulative stress can have on both mental and physical health. It is hypothesized that NET reduces allostatic load thereby fortifying homeostasis and the salutogenic stress response mechanisms involved in recovery from chronic illness, possibly via the Psycho-Immune-Neuroendocrine (PINE) network. Further studies with larger sample sizes are required to establish whether these results could be extrapolated to a wider population, however the results of this case suggest that it may be beneficial to consider co-management of T2D with an MBI such as NET.

## Introduction

This case report describes a novel, non-pharmacological Mind-Body Intervention (MBI) called Neuro-Emotional Technique (NET) that was utilised in the treatment of a 46-year-old male with Type 2 Diabetes (T2D). After 4 weeks of NET treatment, glucose levels stabilized and psychometric tests demonstrated improvements in pain, stress and emotional wellbeing.

### The diabetes epidemic

T2D is a chronic, progressive metabolic condition that occurs when the body becomes resistant to the normal effects of insulin and gradually loses its ability to produce enough insulin in the pancreas, leading to a range of serious physical symptoms and even life-threatening complications (1–6). Even pre-diabetic increases in fasting glucose have been shown to increase all cause risk of death (7), and a diagnosis of T2D is statistically associated with an approximately two-fold increase in mortality, primarily due to cardiovascular complications (8).

Medical literature currently states that there is no known cure for T2D and remission is extremely rare, reported in only about 0.1% of cases (9, 10). T2D affects about 10% of the population and is a major health problem (11). Globally, the incidence of T2D has increased by 70% in recent decades, closely associated with the rising prevalence of obesity and sedentary behaviour (12–16), even in children (12, 17). The estimated economic cost of diabetes in the US alone in 2022 was $412.9 billion and people diagnosed with diabetes have individual health care costs 2.6 times greater than those without (18). The rise of T2D is projected to continue globally from 537 million sufferers in 2021 to 873 million in 2045 (equivalent to about 1 in every 8 adults) (19–21).

### Conventional medical treatment of T2D

The conventional medical management of T2D typically involves a multifaceted approach including life-long education, a balanced diet, frequent exercise, weight management, regular medical check-ups, and continuous blood glucose monitoring, all used as fundamental strategies to help control blood sugar levels (22). When lifestyle modification alone is insufficient, a pharmacological approach involving self-administered insulin injections is typically recommended for life (23). Diabetic pharmacotherapy is aimed at preventing or delaying microvascular and macrovascular complications; however the benefits of anti-diabetic medication may vary depending on patient-specific baseline risk factors (24). While 85.6% of adults diagnosed with diabetes are treated with diabetes medication, only about 50% achieve the target level of glycated haemoglobin (HbA1c), usually due to poor medication adherence (25). The long-term strategy of a conventional medical approach to T2D is a slow intensification of pharmacological therapy “as disease progression advances and β-cell function declines (26)” implying no expectation of improvement, only deterioration.

Mental health symptoms (such as depression) and chronic stress commonly occur in conjunction with T2D and ought to be considered in the management of the condition (27–30). Adverse Childhood Experiences (ACEs) have also been found to strongly correlate with incidence of T2D (31–33), however this association is rarely considered in conventional methods of treatment and prevention (34), representing an area of research that warrants additional investigation.

To our knowledge, no medical literature currently exists to describe the potential effects of NET on T2D. Furthermore, while diabetes research over the last 20 years has focused primarily on the influence of obesity and inflammation (35), comparatively few studies have assessed the influence of non-pharmacological modalities on T2D (for example, Mindfulness-Based Interventions (MBIs) such as yoga, meditation, biofeedback, Mindfulness-Based Stress Reduction (MBSR) etc.), highlighting a need for further contributions to this field. In this case report, therefore NET, a Precision Body-Mind Intervention (PBMI), is presented as a non-pharmacological alternative, or co-management option, for the treatment of T2D.

## Patient information

The personal information of the patient has been de-identified, as per the CARE case report guidelines, and written informed consent was given for the publication of personal health information in print and digital format.

The patient described in this case report is a 46-year-old male reporting high levels of stress due to a demanding workload as a builder and as a father to two children.

The patient had never experienced NET treatment, nor had he utilised any other stress relieving MBIs or non-pharmacological treatment modalities to address his diabetes.

### Clinical findings

Prior to the commencement of the treatment period, the treating practitioner conducted a thorough review of the patient’s medical, family, and psychosocial history.

The patient reported that on May 11, 2023, his blood glucose level was recorded as 28.5 mmol/L and the patient’s general practitioner (GP) provided a diagnosis of Type 2 Diabetes (T2D). [A result of greater than 11.1mmol/L on a random blood sugar test is suggestive of diabetes (36)]. The patient was prescribed and began to self-administer insulin injections. He was also advised by his GP to follow a diabetic eating plan including high fibre / low GI (Glycaemic Index) foods, low salt, minimal sugar and saturated fats, and drinking plenty of water (37). The patient maintained this for two months prior to the treatment period and no other lifestyle or dietary changes (including food type, frequency, or quantity of food intake) occurred during the 4-week NET intervention, or during the 4-week follow up period.

It was noted that the patient wore a Continuous Glucose Monitor (CGM), providing real-time measurements of interstitial glucose as a biofeedback mechanism to assist with insulin self-administration. Despite the use of insulin injections, the patient reported difficulty maintaining glucose levels within the healthy range of 4-10mmol/L on a day-to-day basis.

A review of the patient’s family history revealed that the patient’s mother and uncle (on his mother’s side) have been previously diagnosed with T2D. The patient had sought no additional treatment for stress or diabetes since his diagnosis.

### Primary concerns and symptoms

The patient reported experiencing early symptoms approximately 7-8 months prior to receiving the T2D diagnosis, including anxiety, poor sleep, losing temper easily, dizziness and irrationality. As his symptoms progressed the patient experienced rapid weight loss (from 84kg down to 68kg), insomnia (no more than 4 hours of restless sleep per night), debilitating anxiety, polyuria (needing to urinate 6-8 times every night), constipation, fatigue, physical pain and discomfort, moodiness, and a desire to withdraw from all social contact and activities. The patient described himself as “not a nice person to be around.”

### Examination and assessment

The practitioner performed a postural analysis and physical examination of the spine and musculoskeletal system including range of motion tests and palpation, which were unremarkable. There were no red flags or contraindications identified in the examination and assessment.

As part of the routine intake process for many new patients at this practice, a biopsychosocial assessment was conducted that included the following psychometric tests: ACE-Q (Adverse Childhood Experiences Questionnaire) (38), DASS-21 (Depression, Anxiety and Stress Scale) (39), DRAM (Distress and Risk Assessment Method) (40, 41) and SF-MPQ (Short Form McGill Pain Questionnaire) (42). The results of these tests indicated that this patient was experiencing high levels of chronic stress, distress and pain and had a history of childhood trauma.

### Diagnosis and recommendations

Based on the patient’s health history, the prior medical diagnosis of T2D and clinical findings revealing indicators of chronic stress, the patient was considered a suitable candidate for NET. The practitioner recommended a schedule of 8 treatments at a frequency of two 15-minute sessions per week for 4 weeks. A review of the patient’s progress planned for the 8^th^ visit with a further follow-up assessment conducted 4 weeks later.

The patient fully adhered to the treatment recommendations by attending all 8 treatments and there were no adverse or unanticipated events during the treatment period. There were no barriers to the patient receiving the planned care, nor were there any changes to the therapeutic intervention throughout the treatment period.

### Timeline

In the nine months preceding the NET treatment, the patient retrospectively reported experiencing a gradual worsening of various symptoms. Two months prior, the patient was diagnosed with T2D after returning high serum glucose results, commencing insulin injections and lifestyle changes.

On July 14, 2023, a baseline assessment was conducted, with 24-hour continuous glucose monitor (CGM) readings ranging from 10 to 15 mmol/L, exceeding the healthy range despite two months of consistent lifestyle modifications and insulin use. Baseline measurements were recorded, and the patient commenced NET treatment.

After eight NET treatment sessions, conducted twice per week for four weeks, the 24-hour glucose readings on August 13, 2023, were within the normal range (between 4 and 10 mmol/L). Post-treatment psychometric test results were recorded for comparison with the baseline measurements.

On September 13, 2023, four weeks after the NET treatment concluded, with no further treatment or lifestyle modifications, 24-hour glucose and psychometric tests were assessed again. The patient had attended the funeral of a close friend the day before, and the physiological response to this stressful event may explain the observed afternoon glucose spike. Notably, all psychometric and pain scores stabilized or continued to improve in the four weeks following the NET treatment.

## Therapeutic intervention: the NET treatment protocol

As appropriate, NET is utilised in the treating clinic as a stress-reduction modality where stress plays a significant role as potential underlying factor for autoimmune and/or chronic illness (43).

Neuro-Emotional Technique (NET) is a fast and precise 15-step stress-reduction Mind-Body Intervention aimed at improving physical and emotional health (44). The NET methodology is used to find and remove unresolved, dormant stress patterns in the neurophysiology of the patient called NEC’s (Neuro-Emotional Complexes) (44) that drive adverse bodily responses, potentially contributing to chronic illness (45, 46). NECs are unique to the conditioned, experiential, and emotional reality of each patient and can be quickly identified and relieved, reducing the impact and role of stress that may be underlying the patient’s presenting health condition (44).

To achieve this objective, NET combines elements of numerous health fields, such as pulse assessment in traditional Chinese medicine, cognitive behavioural psychology, gentle spinal adjustments and a reliable feedback method known as the muscle test (47, 48).

Reversing (or extinguishing) classically conditioned painful emotional responses to trauma-related stimuli is a primary objective of the treatment. These stimuli have the unique capacity to replicate a physiological stress response even in the absence of the initial stressor or stressors. The goal of clearing or reducing NEC’s using NET protocols is comparable to exposure therapy and other common cognitive behavioural therapies for severe stress (49).

It is hypothesised that NET works by regulating the homeostatic mechanisms of the Hypothalamic-Pituitary-Adrenal (HPA) Axis and the Psycho-Immune-Neuroendocrine (PINE) Network (50–54) thereby relieving allostatic load, as explored further in the Discussion.

Published research demonstrating these effects includes an RCT where NET was shown to lower inflammatory blood markers and relieve pain in a cohort of chronic low back pain sufferers (55), and another revealed reduced activity in brain areas involved with traumatic memories and distress (such as the anterior cingulate gyrus, parahippocampus, insula and brainstem) in a cohort of cancer survivors (56). For a more detailed description of NET we refer the reader to previously published detailed explanations of the technique as applied in standardised clinical research settings (57–62).

## Results

The patient experienced relief of all symptoms reported including a reduction of physical pain/discomfort as well as a decrease in mood dysregulation (anxiety, irritation, losing temper, irrationality etc.) He reported normalisation of sleep and nocturia, and his symptoms of anxiety, fatigue, constipation and dizziness ceased. The results of the psychometric tests, self-reported pain assessment and interstitial glucose measurements are as follows:

### The adverse childhood experiences questionnaire

The Adverse Childhood Experiences (ACE) Questionnaire is a popular assessment instrument made to gauge the severity of adversity in childhood and its potential to affect a person's physical and mental health in adulthood (38). The patient in this case report returned a score of 4 on the ACE-Q, which lies within what we call “the auto-immune risk zone” because any ACE score greater than 2 represents a 70-80% risk of experiencing an auto-immune condition in adulthood (63), including a strong correlation with a predisposition to T2D (31–34).

### The depression, anxiety and stress scale

The DASS-21 (Depression, Anxiety and Stress Scale) Questionnaire is a popular psychometric tool for assessing and quantifying the severity of a person's symptoms of depression, anxiety, and stress (64). It is a reliable self-report questionnaire that enables respondents to provide information about their emotional health (39, 65). Higher scores in each of the three categories indicate symptoms that are more serious in nature (64).

Five levels of severity, from “normal” to “extremely severe”, are utilised in the interpretation of DASS-21 results. “normal” scores are: Depression (<9), Anxiety (<7) and Stress (<14). “Extremely severe” scores are: Depression (28+), Anxiety (20+) and Stress (34+). People with an "extremely severe" score on the DASS-21 are likely dealing with severe psychological symptoms that may seriously impair their ability to operate on a day-to-day basis, as well as their general wellbeing and quality of life (39).

**Figure 1:**
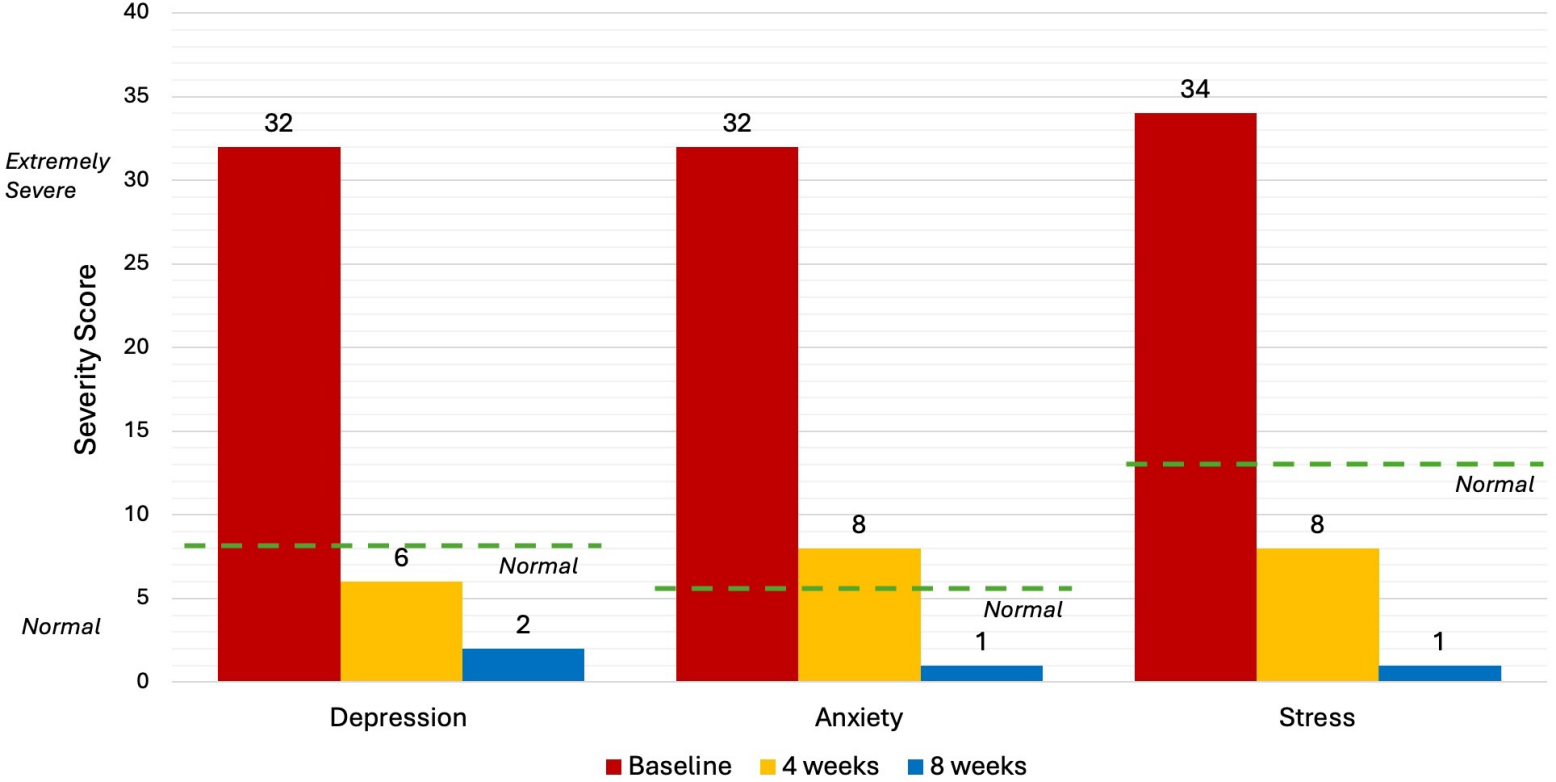
DASS-21 Results at Baseline, 4 weeks and 8 weeks.

Upon commencement of the treatment, DASS-21 scores for this patient were “extremely severe” in each of the three categories: Depression (32), Anxiety (32) and Stress (34). After 8 NET sessions (4 weeks) the patient completed a second DASS-21 evaluation and returned “normal” results in each category. After 8 weeks, the scores in each category further decreased indicating continued improvement after the treatment period (**Figure 1**).

### The distress and risk assessment method

The DRAM (Distress and Risk Assessment Method) is a practical means of assessing the level of psychological distress in patients with pain (40, 41). It is a validated measuring tool consisting of two questionnaires, the MZDI [Modified Zung Depression Index (66)] and the MSPQ [Modified Somatic Perception Questionnaire (67)]. The MZDI is designed to give a quantitative measure of a person’s level of depression while the MSPQ is a measure of somatic awareness and anxiety, with the combined results of the MZDI and the MSPQ tallied to provide a DRAM score.

Typically, a high DRAM score is considered a poor predictor of treatment success (68). The four DRAM classifications as per Main et al. (40) are: Normal = MZDI < 17; At Risk = MZDI 17-33 and MSPQ < 12; Distressed Depressive = MZDI > 33 and Distressed Somatic = MDZI > 33 + MSPQ > 12 (40).

**Figure 2:**
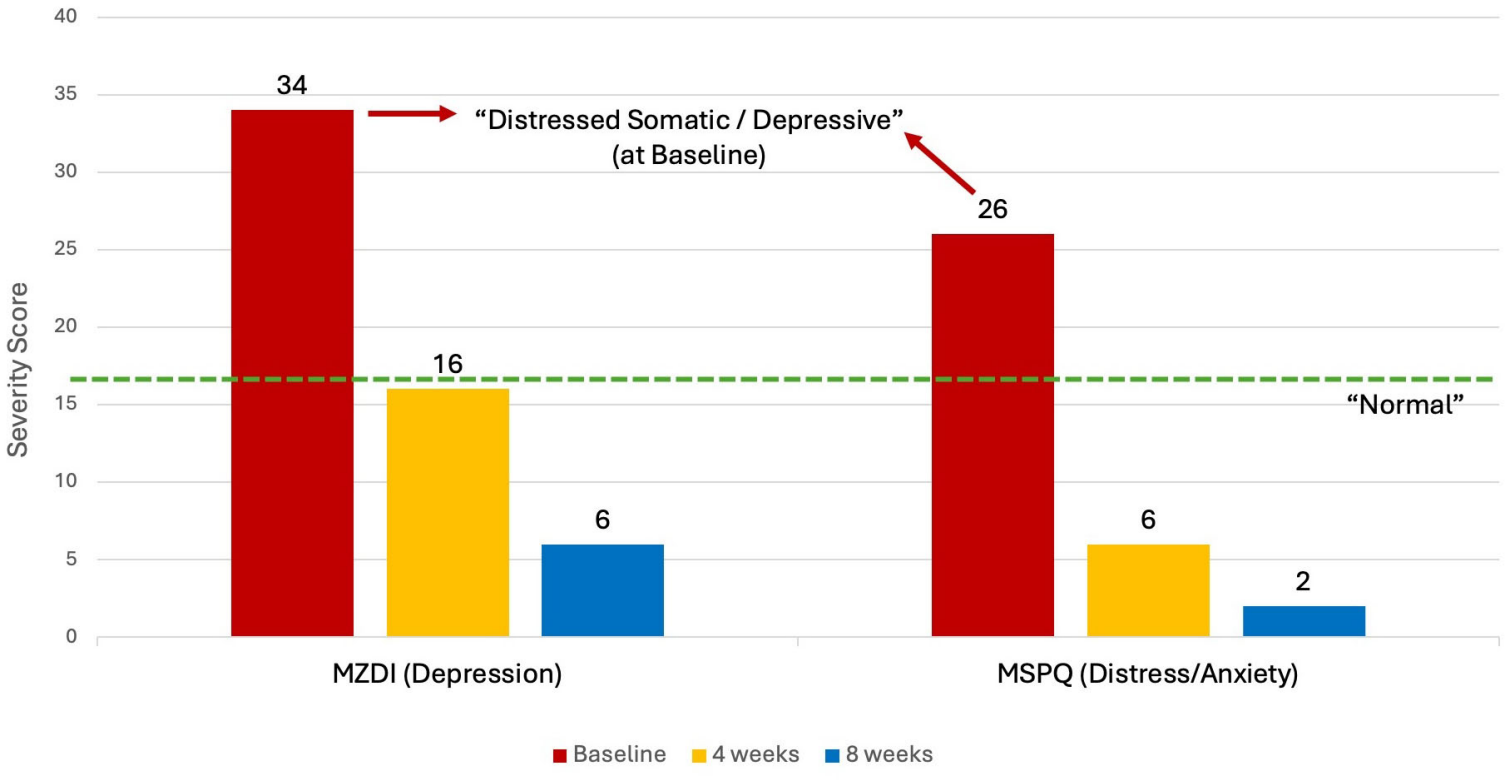
DRAM at Baseline, 4 weeks and 8 weeks. DRAM, Distress and Risk Assessment Method; MZDI, Modified Zung Depression Index; MSPQ, Modified Somatic Perception Questionnaire.

The baseline DRAM scores for the patient in this case report were an MSPQ (distress) of 26 and a MZDI (depression) of 34, resulting in a classification of “Distressed Somatic/ Depressive”. The MSPQ (distress) score dropped to 6 and then 2 after 4 and 8 weeks respectively. The MZDI dropped from 34 to 16 and then 6 at the same intervals. The patient’s overall DRAM classification changed to “Normal” after 4 weeks of NET treatment, with further improvements noted at 8 weeks (**Figure 2**).

### The short form McGill pain questionnaire

The Short Form McGill Pain Questionnaire (SF-MPQ) is a reliable and valid assessment of a person’s intensity and frequency of physical discomfort (42). It includes four primary components: Present Pain Intensity (PPI) scale, the Visual Analogue Scale (VAS) and 15 pain descriptors (11 Sensory, 4 Affective) that can be scored on an intensity scale where 0 = none, 1 = mild, 2 = moderate and 3 = severe.

**Figure 3:**
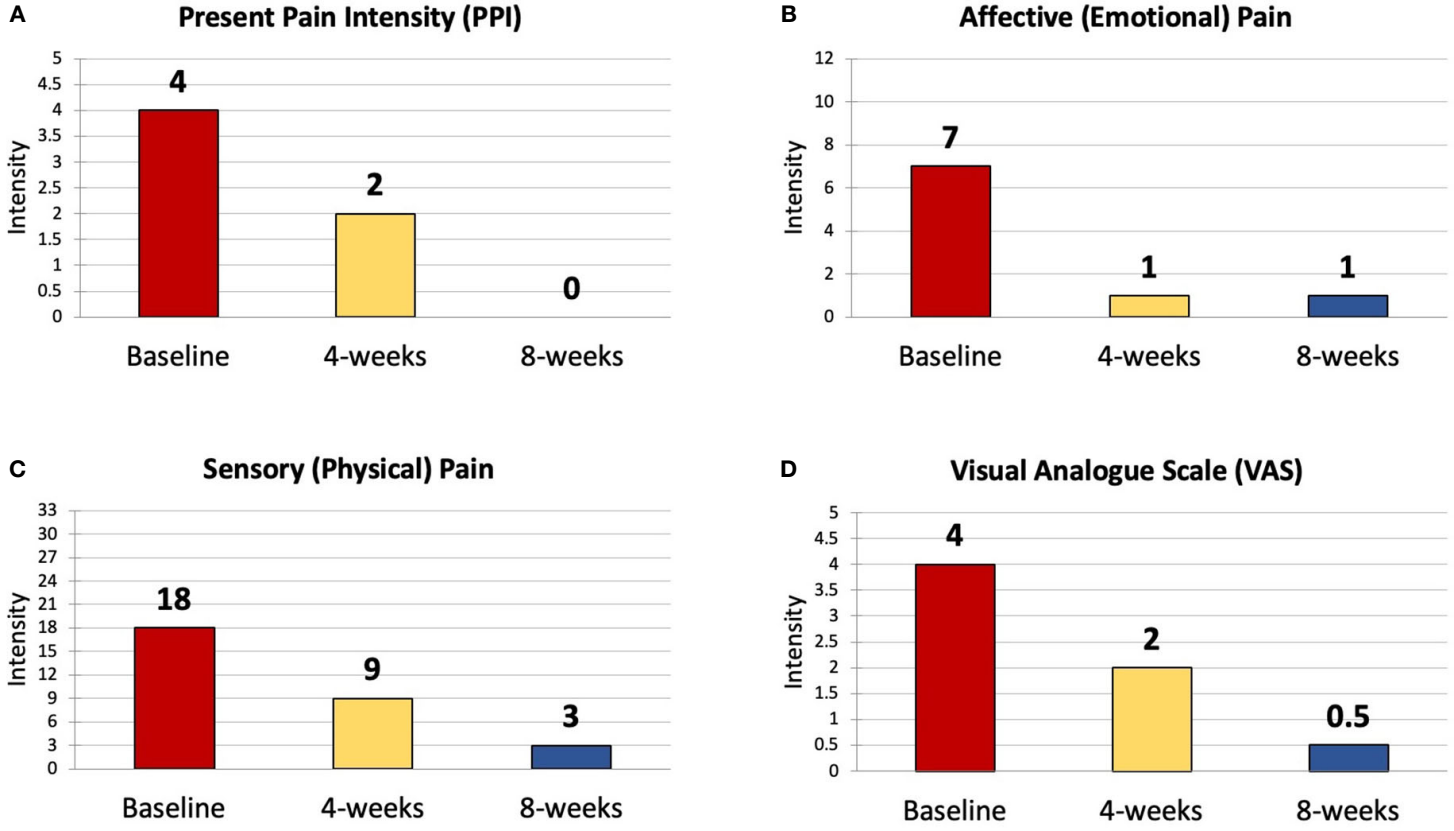
The Four Components of the SF-MPQ - Results at Baseline, 4 weeks and 8 weeks. SF-MPQ, Short Form McGill Pain Questionnaire; PPI, Present Pain Intensity; VAS, Visual Analogue Scale. **(A)** Present Pain Intensity (PPI); **(B)** Affective (Emotional) Pain Intensity; **(C)** Sensory (Physical) Pain Intensity; **(D)** Visual Analogue Scale (VAS) Pain Rating.

The results of the SF-McGill showed a 100% decrease in Present Pain Intensity (PPI) from a score of 4/5 to 2/5 at 4 weeks to 0/5 at 8 weeks (**Figure 3A**). Affective (emotional) pain dropped by 85% from 7/12 to 1/12 at 4 weeks and this improvement was sustained at 8 weeks (**Figure 3B**). Sensory (physical) pain was reduced by 80% from 18/33 to 9/33 at 4 weeks and further decreased to 3/33 at 8 weeks (**Figure 3C**). The Visual Analogue Scale (VAS) fell from 4/5 to 2/5 at 4 weeks and 0.5/5 at 8 weeks (**Figure 3D**).

### Interstitial glucose

**Figure 4:**
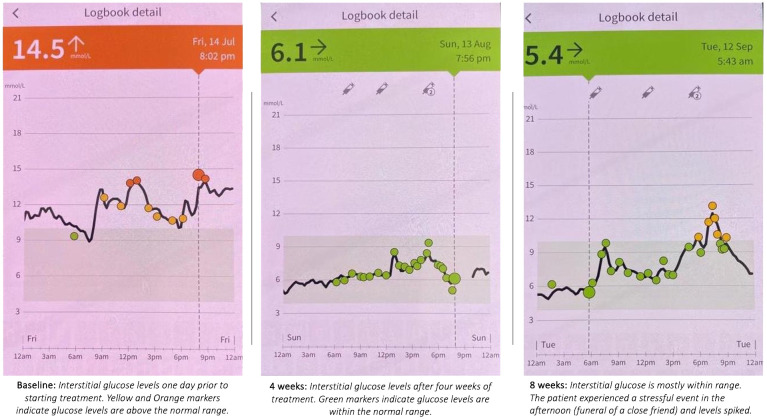
Interstitial Glucose at Baseline, 4 weeks and 8 weeks .

The patient wears a continuous glucose monitoring (CGM) sensor called “FreeStyle Libre 2” which is a medical device registered with the Therapeutic Goods Administration (TGA) in Australia (69). The sensor is placed on the patient’s skin allowing constant detection of glucose levels in the Interstitial Fluid (ISF). ISF glucose levels are comparable to and follow blood glucose levels after a period of time known as “the lag” (70). Interstitial glucose levels are transmitted to the FreeStyle LibreLink app on the patient’s mobile device. Data from the sensor was gathered from the patient’s phone to compare ISF glucose levels at baseline and then after 4 and 8 weeks of treatment (**Figure 4**).

The optimal zone for glucose levels is between 4.0-7.0mmol/L (fasting) and 5.0-10.0mmol/L (postprandial) (71). Prior to commencing treatment, the patient’s baseline levels were above the healthy range for almost the entire 24-hour period (**Figure 4**, Baseline). After four weeks of treatment, glucose measurements across a full 24-hour period are entirely within the optimal range 4.0-10mmol/L. At 8 weeks interstitial glucose remains mostly within the optimal range, however there is a spike above optimal levels late in the day following a stressful event - the funeral of a close friend. This rise in glucose levels practically demonstrates a connection between biopsychosocial stress and subsequent adaptation of the PINE network, as explored in the Discussion.

## Discussion

Considering the anticipated global rise of T2D, and the mixed success and poor prognosis of the conventional medical approach to treatment, it is appropriate to investigate the potential benefits of adjunct or alternative treatments (such as MBIs) that may better address the underlying physiological dysfunction and chronic stress at the root of the condition.

### Outcomes of MBIs on T2D

MBIs commonly examined in the scientific literature as potential treatments for T2D include mindfulness training programs such as Kabat-Zinn’s MBSR (Mindfulness-Based Stress Reduction), Mindful Awareness Programs (MAP’s), Cognitive Behavioural Therapy (CBT) and Medical Nutrition Therapy (MNT) (72–74) but may also include more dynamic, movement-based practices such as yoga, tai chi and qigong (75). MBIs have been found in systematic reviews to not only reduce psychological stress, but also induce physiological changes including parasympathetic activation, lower cortisol secretion, down-regulate pro-inflammatory genes and pathways, delay the rate of ageing, counter the effects of chronic stress, and reduce blood glucose, which are all risk factors for T2D (76).

However, benefits vary greatly between interventions and there is significant heterogeneity between studies making it difficult to generalise the effects, influences and cost-effectiveness of MBIs as a whole (75, 77). For example, one systematic review found that in older, working-aged adults MBSR may help reduce participants’ perception of depression immediately following the intervention, but there paradoxically appeared to be no effect on perceived levels of anxiety or stress (78). Moreover, a systematic review of Randomised Controlled Trials (RCTs) assessing the efficacy of psychological interventions on T2D showed no conclusive improvements in levels of Diabetes Related Distress (DRD) or Health Related Quality of Life (HRQoL), with only slight improvements in diabetic blood markers after a median treatment duration of 6 months (79). Another literature review of MBSR research studies ironically revealed no conclusive evidence of lasting stress reduction beyond the treatment period and did not detect effects on the diabetic blood marker HbA1c (80). Authors Hackett and Steptoe identified that stress-relieving MBIs improved symptoms of depression and DRD but a positive effect on glycaemic control was less certain across a range of studies (30), however Yang et al. identified several studies showing “MBIs can significantly contribute to blood glucose control” (76). Future studies with larger cohorts over longer time periods may help to clarify some of these inconsistencies.

### Allostatic load and T2D

Interestingly, the NET treatment evidenced in this case report resulted in consistent improvements across a range of measurable psychological markers *and* lowered glucose levels, all within a comparatively short treatment period of just 4 weeks. We hypothesise that this is due to the precise physiological reduction of dormant emotional stressors facilitated specifically by the NET treatment, thereby lowering the allostatic load that may have been a significant contributor to the accumulated physiological dysfunction that eventually led to the manifestation of T2D (30).

McEwen’s allostatic load concept refers to the *cumulative* burden of chronic stress and challenges from life events, and the resultant difficulty for the body to return to homeostasis (81). Dysregulation of glucose metabolism, neuroendocrine function, diurnal cortisol release and chronic low-grade inflammation (LGI) can result via disruption of the PINE Network, thereby contributing to the development of chronic conditions such as T2D (30). Emerging research suggests that both T1D and T2D may express aspects of autoimmunity [loss of self-tolerance (82)] primarily due to pervasive LGI, both in the pancreas and system-wide throughout the body (83, 84). Alongside metabolic dysregulation, a vicious cycle then results: increasing cytokine (inflammatory cell) production destroying pancreatic β-cells, in turn leading to the release of autoimmune “self” antigens that further impair insulin secretion and promote hyperglycaemia, triggering even more inflammation (85, 86). Understanding what can cause allostatic load to chronically increase beyond physiological tolerance, setting off these metabolic and immune disease processes, may inform an understanding of why a stress relieving MBI like NET can benefit T2D.

### Adverse childhood experiences and T2D

Allostatic overload can occur due to micro-stressful events (MSEs) such as chronic work stress, financial pressure, health crises such as depression, or the presence of persistent and unresolved early life stressors (ELS), such as those quantified in the ACE-Q. People who report four or more ACE’s are statistically more likely to report chronic health conditions compared to those who report experiencing zero adverse childhood events (32, 87–91). ELS and Adverse Childhood Experiences (ACEs) can predispose sufferers specifically to T2D (34, 92–94), especially the presence of any of the following: undergoing childhood economic adversity, suffering abuse (verbal, physical or sexual) or having a family member incarcerated (33). The addition of a single ACE equates to an approximately 11% increase in the odds of diabetes (95) and an ACE-Q score of 4 or more is associated with a 2.1 times greater risk of diabetes (31).

### Stress, NET and T2D

A review of the physiological response to chronic stressors, such as unresolved ELS or childhood mistreatment (CM), may explain these associations between ACE scores and increased risk of T2D (96). Initially, glucocorticoid hormones, such as cortisol, are released by the adrenal medulla in response to a stressor, increasing insulin secretion (97). Glucocorticoids influence glucose homeostasis by inducing the release of glucose and lipids into the circulation, in turn activating the Sympathetic Nervous System (SNS). The SNS acts then to release adrenaline, increasing heart rate and blood pressure, lowering heart rate variability, mobilising energy and releasing pro-inflammatory cytokines. Repeated or sustained stimulation of the allostatic system can cause an overabundance of glucose and lipids relative to the cellular energy demands, constituting a form of metabolic stress that can promote insulin resistance, weight gain and an amplified immune reactivity due to the presence of heightened inflammation (30, 83, 98). The “Biological Embedding of Childhood Adversity” model has been proposed to explain how childhood stress becomes epigenetically “programmed” into macrophages, consequentially endowing cells with pro-inflammatory tendencies, thereby exaggerating cytokine responses and decreasing sensitivity to inhibitory hormone signals, altering endocrine and autonomic patterns that, in conjunction with resulting genetic and lifestyle influences, ultimately results in chronic inflammation that fosters chronic disease (99).

Inflammation is both a key marker of chronic stress and a contributing factor in the pathophysiology of T2D (100). CRP (C-Reactive Protein), a blood marker for inflammation, is known to independently elevate in adults 20 years after experiences of childhood maltreatment or trauma (101). The assessment of ACEs in this case report is appropriate and informative because increased serum CRP levels not only predict the risk of T2D, small increases in CRP can also predict the likelihood of cardiovascular events – a common comorbidity of T2D (102). NET can be appropriately considered as a treatment option because the technique has been shown in a RCT by two of the authors to reduce CRP levels, as well as other inflammatory blood markers associated with stress [such as tumour necrosis factor-α (TNF-α), interleukin-1 (IL-1), and IL-6 (55)], all of which can be predictive of T2D (100).

The resolution or reduction of dormant stressors for a patient with unresolved childhood trauma – and an associated decrease in stress-induced inflammation and allostatic load - may, at least in part, explain the effectiveness of the NET treatment in this case report. A similar treatment effect has been proposed in a published case report of a hypothyroidism patient with a high ACE score who demonstrated normalisation of thyroid blood markers and psychometric stress following NET treatment (54).

### Strengths and weaknesses

A weakness of this case report is that the treatment was performed by a single practitioner on a single patient, therefore the results cannot yet be generalised for other practitioners, or to a wider population of T2D sufferers. It is possible that NET may not be a suitable intervention or co-management strategy for all sufferers of T2D. Another weakness is that additional data from the patient’s CGM was not available for publishing, making it difficult to ascertain further insights into the precise mechanisms and future trends associated with the patient’s response to treatment.

Strengths of this case report include reliable outcome measures with pre- and post-treatment changes that could help to establish protocols for larger RCT based studies. In future studies, more pre- and post-treatment CGM data (for example, “average glucose” and “time in range”), measures of additional blood markers such as Fasting Blood Glucose, Fasting Insulin, HbA1c, inflammatory cytokines such as (TNF-α, IL-1β, IL-6, IL-8 and CRP) and the assistance of an experienced biostatistician could be garnered for deeper insights into the physiological responses to NET treatment.

Should the results of this case report be reproduced in longitudinal, randomised controlled trials with a greater number of patients and practitioners involved, NET may be identified as a reliable co-management intervention to assist sufferers of adverse childhood events and chronic stress-based illnesses such as T2D.

## Conclusion

This case report describes the effect of a new, stress-relieving MBI called NET on psychological and physiological markers of T2D, resulting in normalisation of symptoms, stress, and glucose levels over a 4-week treatment period. The changes in objective and subjective measures (including reduction in physical pain, psychological distress and interstitial glucose levels) were sustained, and many indicators showed continued improvement, 4 weeks after the treatment period concluded. The allostatic load created by early life stress (ELS) – represented by a high score on the Adverse Childhood Experiences (ACE) questionnaire that correlates with increased likelihood of auto-immune disorders such as Type 2 Diabetes (T2D) – may have been discharged by the stress-relieving and inflammation-lowering effects of the NET treatment. In similar cases where conventional pharmacological management alone is insufficient for moderating symptoms and blood glucose levels associated with T2D, NET may prove to be a valuable co-management strategy to address underlying physiological stressors that exacerbate or cause the condition.

## Patient perspective

“After receiving this treatment for my Type 2 Diabetes, my life has changed in many positive ways. Mentally, I feel I can cope much better with everyday life and its challenges. Mentally and emotionally, I feel "reset", a lot more positive and less foggy than I did before treatment. I would describe my experience of the treatment I received as pivotal, because it helped me change my mind-set about my condition and more positive as a result. I feel acceptance and accepting that I now have to balance my insulin injections & food intake. This has given me relief, as well as provided me with the confidence that I am able to manage and regulate my condition. I realise that I now prioritise health and lifestyle as much as I prioritise work. All around, I am happier with myself, and more balanced.”

## Data availability statement

The original contributions presented in the study are included in the article/supplementary material. Further inquiries can be directed to the corresponding author.

## Ethics statement

Written consent was received from the patient for the publishing of the case report. The studies were conducted in accordance with the local legislation and institutional requirements. The participants provided their written informed consent to participate in this study. Written informed consent was obtained from the individual(s) for the publication of any potentially identifiable images or data included in this article.

## Author contributions

PB: Conceptualization, Data curation, Methodology, Project administration, Writing – review & editing. RD: Writing – original draft, Writing – review & editing. HP: Supervision, Writing – review & editing.
